# Cytokine profile in subjects with Cystic Fibrosis and nasal polyposis compared to patients with no nasal disorders

**DOI:** 10.1590/S1808-86942010000100005

**Published:** 2015-10-17

**Authors:** Flávio Barbosa Nunes, Mirian Cabral Moreira de Castro, Tacimara Moreira da Silva, Ricardo Nascimento Araújo, Helena Maria Gonçalves Becker, Paulo Fernando Tormin Borges Crosara, Roberto Eustáquio Santos Guimarães

**Affiliations:** 1 PhD in Medicine, HFMG; Substitute Professor, Department of Ophthalmology and Otorhinolaryngology, UFMG; 2 MSc in General Surgery, UFMG Medical School; Advisor, Medical Residency Service, UFMG University Hospital and Santa Casa de Misericórdia Hospital - MG; 3 MSc in General Surgery, UFMG Medical School; MD, ENT, General Surgeon, Medical Officer at the Minas Gerais Military Police; 4 PhD in Parasitology, UFMG; Adjunct Professor, Department of Parasitology, Federal University of Minas Gerais; 5 PhD in Ophthalmology and Otorhinolaryngology, UFMG; Adjunct Professor, Department of Otorhinolaryngology and Ophthalmology, UFMG Medical School; 6 PhD in Ophthalmology and Otorhinolaryngology, UFMG; Adjunct Professor, Department of Otorhinolaryngology and Ophthalmology, UFMG Medical School; 7 PhD in Medicine; Professor, USP Medical School at Ribeirão Preto; Adjunct Professor, Department of Otorhinolaryngology and Ophthalmology, UFMG Medical School

**Keywords:** cytokines, cystic fibrosis, nasal polyps

## Abstract

Although the cytokine profile in nasal polyposis is well documented, little is known about cytokines associated to cystic fibrosis.

**Aim:**

Assess the expression of cytokines IL⌝4, IL⌝5, IL⌝6, IL⌝8, GM⌝-CSF and IFN⌝-y, analyzed through RT-PCR, in the polyps of patients with cystic fibrosis.

**Materials and Methods:**

A cross-sectional, prospective study was carried out with 24 patients, 13 of whom had cystic fibrosis and nasal polyposis (Cystic Fibrosis Group) and 11 had normal otorhinolaryngological exams (Control Group). The average age was 21 years (3⌝57); 12 participants were males and 12 were females. The cytokine profile was studied in mucosal fragments (Control Group) or nasal polyps (Cystic Fibrosis Group) through RT⌝PCR. Transcriptions were studied for cytokines IL⌝4, IL⌝5, IL⌝6, IL⌝8, IFN⌝y and GM⌝CSF, adjusted for the ß⌝-actin value.

**Results:**

Interleukins 5, 6, 8 and GM⌝CSF were similar in both groups (p>0.05). There were lower values of IFN-y⌝ (p=0.03) and a strong tendency toward an increase in IL⌝4 (p=0.06) in the Cystic Fibrosis Group.

**Conclusion:**

Inflammatory and structural cells may produce messenger RNA for IL⌝4, blocking the production of other cytokines such as IFN-y, suggesting the participation of this mechanism in the formation of polyps in cystic fibrosis.

## INTRODUCTION

Nasal polyposis (NP) is a chronic inflammatory disease that involves the nasal mucosa. It is characterized by the presence of multiple grayish nasal polyps located bilaterally and originating from the middle meatus^1^,^2^. NP involves 0.5% of the population; eosinophilic nasal polyposis (ENP) accounts for 85% of the cases, while non-eosinophilic nasal polyposis (NENP) accounts for the remaining 15%^1^,^2^.

Diseases with concurrent NENP include mucovisci-dosis or cystic fibrosis (CF). This is a congenital autosomal recessive disease found in 1 of every 2,500 individuals. Patients experience exocrine gland dysfunction, chronic pulmonary obstruction, and pancreas failure. Polyps are present in 20% of the patients^3^,^4^.

Various pathogenic mechanisms have been proposed to explain its development, among which are allergy and inflammation. Although the genesis of allergic rhinitis is IgE-specific, its prevalence is of only 1.5% of the patients with polyposis, and similar levels of immunoglobulin E were observed in polyps of both allergic and non-allergic patients^5^.

The inflammatory micro-environment, cytokines, adhesion molecules, and ion transportation have been recently studied to clarify the pathogenesis of nasal polyps^6^.

More specifically, eosinophils and mediators such as interleukins 4 (IL-4), 5 (IL-5), 6 (IL-6), 8 (IL-8), granulocyte-macrophage colony-stimulating factors (GM-CSF) and interferon gamma (IFN-γ), were given special attention after elevated values were observed in patients with ENP1.

Unfortunately, few papers have looked into NP and the role of cytokines in CF.

Therefore, this study aims to analyze the expression of messenger ribonucleic acid (mRNA) for IL-4, IL-5, IL-6, IL-8, GM-CSF, and INF-γ in patients with cystic fibrosis and control subjects through reverse transcription polymerase chain reaction (RT-PCR).

## MATERIALS AND METHOD

This is a cross-sectional study done on 13 patients with NP and 124 patients with CF. Six (46.15%) of the 13 patients were males and seven (53.85%) were females with mean age of 22.45 years (6–57).

The following criteria was used to enroll and exclude subjects from the study.

### Enrollment criteria

Diagnosis of CF based on two dosages of electrolytes in sweat with sodium and chlorine levels above 60mEq/l.

Patients with CF and NP.

### Exclusion criteria

Upper or lower airway infection, use of topical or systemic glucocorticoids or antihistamines within 30 days of the study.

The control group was made up of 13 individuals who looked for our service to undergo ENT surgery. Nasal examination did not show any alterations for these subjects, and allergies were ruled out after dermal puncture tests^7^; none of them was taking topical/systemic glucocorticoids or antihistamines. Two patients were excluded due to immediate postoperative acute sinusitis. From the remaining eleven, five (45.45%) were females, six (54.54%) were males, with a mean age of 17.7 years (3–43).

The study was carried out from December, 2003 through December of 2004, and was approved by the Ethics in Research Committee of the University of Minas Gerais Medical School Hospital, under protocol # n^o^. 209/03. All the patients signed the post-informed consent form to participate in this study.

The cytokine profile was analyzed looking at mucosa fragments from the middle turbinate (control group) and polyp specimens (cystic fibrosis group) to collect mRNA for further analysis using RT-PCR.

Polyp fragments from the CF group were collected at the ENT outpatient ward using an EXPLORENT® forceps (Karl Storz, Miami, Florida, USA) and a Storz 30^o^, 4.0 mm endoscope (Karl Storz, Miami, FL, USA).

Normal nasal mucosa fragments from control group patients were collected in the middle turbinate using an EXPLORENT® forceps (Karl Storz, Miami, FL, USA) and a Storz 30^o^, 4.0 mm endoscope (Karl Storz, Miami, FL, USA) during ENT surgical procedures. Patients were under general anesthesia and the collected specimens were sent for further testing using RT-PCR.

Transcriptions for cytokines IL-4, IL-5, IL-6, IL-8, GM-CSF, and INF-γ were analyzed and adjusted by the β-actin coefficient8. After cooling the samples to −80°, RNA extraction was done using reagent Trizol® (Invitron Corporation, Carlsbad, California, USA) in accordance with the manufacturer's recommendations. RNA concentration in the samples was quantified in a spectrophotometer for the wavelength of 260 nm and integrity was assessed through denaturating gel electrophoresis using 0.8% agarose^8^. Deoxyribonucleic acid (DNA) was synthesized from 1.25μg of total RNA using random hexamer initiators (Promega Corporation, Madison, WI, USA) and reverse transcriptase system SuperScript II (Invitron Corporation, Carlsbad, CA, USA) as recommended by the manufacturers. PCR products were analyzed through silver-stained 8% polyacrylamide gel electrophoresis. The gels were photographed and results analyzed through densitometry using device Alpha-Digidoc 1201 TM (AlphaInotech, San Leandro, CA, USA). The resulting PCR bands for each sample were analyzed through densitometry using program AlphaEaseFC release 3.3.0 (AlphaInotech, San Leandro, CA, USA).

ANOVA (analysis of variance) parametric tests and Student's T-test were used in the statistical analysis to compare two mean values and 5% was adopted as statistical significance level.

## RESULTS

Table 1 shows that no statistically significant differences were found when IL-5, IL-6, IL-8 and GM-CSF values were compared between groups (p>0.05).

**Table 1:** Comparison between age and cytokine level adjusted for β-actin.

l Group					Cystic Fibrosis Group				
Variable	Mean	SD	Median	Amplitude	Mean	SD	Median	Amplitude	p-value
IL-4	38538,0	21015,0	36495,9	12558,3–82459,7	64287,5	32835,1	56187,1	20064,7–104356,6	0,06
IL-5	27803,6	15974,8	23625,3	6444,0–57867,5	29918,5	22119,2	28308,6	4328,7–81327,9	0,88
IL-6	60259,9	52404,5	40113,7	15571,0–175675,4	60631,7	34630,0	67502,5	6905,3–112570,8	0,51
IL-8	66254,5	47421,5	53019,9	13326,8–171973,0	74032,6	27356,4	78545,1	24567,5–120438,6	0,40
IFN-γ	153510,3	65026,5	126851,7	80603,1–281112,5	100839,3	40426,2	94889,7	48449,2–190901,9	0,03
GM-CSF	4958,1	1306,4	5286,0	1602,2–6455,9	4119,6	2627,1	3033,9	918,1–8446,1	0,40
Age	17,7	13,6	15,0	3–43	21,1	12,5	20,0	6–57	0,32
β-actina	130960	22315	154549	90090–174528	150324	23318	135790	103700–179450	0,052

SD = standard deviation.

Table 1 also shows higher values for IL-4 (p=0.06) strongly tending to statistical significance and low values for INF-γ (p=0.03) in the cystic fibrosis group when compared to the control group.

## DISCUSSION

Cytokines are secreted by inflammatory cells (lymphocytes, eosinophils, and neutrophils) and constituting cells (mastocytes, fibroblasts, epithelial cells, and macrophages)^9^. They regulate biologic processes such as growth, cell activation, chemotaxis, inflammation, immunity, tissue repair, fibrosis, and morphogenesis^10^. The profile of cytokines and inflammatory cells in ENP is well documented in the literature; conversely, very little is known on the pathogenesis of NP and cytokines in CF patients.

Interleukin 8 is the main cytokine related to NP in CF patients. Various authors have observed increased levels of IL-8 and neutrophils when comparing such patients to subjects with ENP^11^. The main sources of IL-8 are neutrophils and their byproducts such as elastase, oxidants, and IL-8^12^,^13^.

Sobol et al.^12^ looked at individuals with chronic sinusitis and CF and compared them to healthy subjects and showed that nasal disease in CF consists of a neutrophilic infiltrate with increased levels of IL-8, similarly to what is found in patients with lung disorders^12^.

It is worth mentioning that, similarly to what was found in this study, Noah et al.^14^ and Black et al.^15^ showed that increased IL-8 levels are consequent acute associated bacterial infection. In the absence of infection - infection being one of the exclusion criterion in this study - both nasal and lung secretions showed normal IL-8 levels^14^,^15^.

The respiratory epithelium impacts polyp inflammatory mediation, mainly in ENP, through the production of cytokines such as IL-6 and GM-CSF^16^,^17^. Thus, differently from Bernstein et al.^6^ and Mulloi et al.^16^ in which large epithelial lesions and consequent increases in GM-CSF and IL-6 were respectively shown, for nasal polyps this study showed a wide range of normal values for such cytokines, suggesting greater epithelium integrity on NP in CF patients and a lesser participation of IL-6 and GM-CSF in this micro inflammatory environment.

Eosinophils had genetic expression for IL-5 in patients with ENP^10,^^18,^^19^.

According to Jankowski et al.^20^, eosinophils, even at lower levels as found in NP CF subjects, play an important role in inflammation in the development of polyps^20^.

As indicated on Table 1, no increases were seen on IL-5 as similarly found by Clayes et al.^4^ and Sobol et al.^12^. These results suggest that if eosinophils have an important role in NP associated with CF, different mechanisms exist for eosinophilia and are independent from IL-5.

Interestingly enough, as seen on Table 1, we found that increased levels of IL-4 tended towards statistical significance and low levels of INF-γ in the cystic fibrosis group.

Similarly as found in our study, Clayes et al.^4^ and Dellacono et al.^21^ showed lower levels of INF-γ in patients with CF and nasal polyposis when compared to subjects with eosinophilic polyps. INF-γ was increased in CF patients only in acute sinusitis events^12^.

In contrast, we could not find papers in the literature showing altered IL-4 values in patients with nasal polyposis and cystic fibrosis.

Bacterial lipopolysaccharides (LPS) bind to the receptor of CD4+ T-cells (T-helper 2 cells) and stimulate the production of IL-4, leading to increased IgE values^22^, ^23^, ^24^. By its turn, INF-γ introduces an opposite effect through stimulation of T-helper 1 cells (Th1) and by blocking the production of IgE and T-helper 2 cells (Th2), principally IL-4 and IL-5^24^.

Originally defined as an antiviral agent, INF-γ plays an important role in stimulating bactericide action by macrophages, antigen presentation through MHC (major histocompatibility complex) molecules, and leukocyte-en-dothelium interaction^25^,^26^. It suppression leads to bacterial proliferation due to the absence of intracellular clearance or by epithelial ionic alterations^25^,^26^.

Thus, reductions on INF-γ levels as observed in this study would explain the presence of bacteria or bacterial LPS in the intracellular realm. These bacteria would stimulate the production of IL-4 by Th2 cells. The outcome would be intense inflammation with increased levels of eosinophilic cationic protein (ECP), total IgE, led by increased levels of IL-4 and reduced levels of INF-γ^23^,^24^.

Therefore, Staphylococcus aureus, Pseudomonas aeruginosa or bacterial LPS could induce eosinophilic inflammation and the synthesis of polyclonal IgE, elevating total IgE levels led by increased IL-4 levels and reduced levels of INF-γ^23^,^24^. Enteroxin would thus modify nasal polyposis through the IgE released by mastocytes and would act as an alternative mechanism to allergy^24^.

## CONCLUSION

The expression of mRNA for interleukins 5, 6, 8, and GM-CSF analyzed through RT-PCR was similar among the control and cystic fibrosis groups. Low INF-γ levels and tendency to increased IL-4 levels were found in the group with cystic fibrosis.
